# Hepatic Steatosis Analysis in Metabolic Dysfunction-Associated Steatotic Liver Disease Based on Artificial Intelligence

**DOI:** 10.3390/diagnostics14242889

**Published:** 2024-12-23

**Authors:** Xiao-Xiao Wang, Yu-Yun Song, Rui Jin, Zi-Long Wang, Xiao-He Li, Qiang Yang, Xiao Teng, Fang-Fang Liu, Nan Wu, Yan-Di Xie, Hui-Ying Rao, Feng Liu

**Affiliations:** 1 Peking University People’s Hospital, Peking University Hepatology Institute, Infectious Disease and Hepatology Center of Peking University People’s Hospital, Beijing Key Laboratory of Hepatitis C and Immunotherapy for Liver Diseases, Beijing International Cooperation Base for Science and Technology on NAFLD Diagnosis, Beijing 100044, China; wangxx0635@163.com (X.-X.W.); p@k3b4.onmicrosoft.com (Y.-Y.S.); 18810531268@163.com (R.J.); zeelomwang@bjmu.edu.cn (Z.-L.W.); lxhe0903@163.com (X.-H.L.); wn_summer@163.com (N.W.); xieyandee@163.com (Y.-D.X.); rao.huiying@163.com (H.-Y.R.); 2 Hangzhou Choutu Technology Co., Ltd., Hangzhou 310052, China; yang.qiang@choututech.com; 3 HistoIndex Pte Ltd., Singapore 117674, Singapore; teng.xiao@histoindex.com; 4 Department of Pathology, Peking University People’s Hospital, Beijing 100044, China; reddove_hg@aliyin.com

**Keywords:** non-alcoholic fatty liver disease, mouse models, steatosis, second-harmonic generation (SHG)/two-photon-excited fluorescence (TPEF)

## Abstract

Background: Metabolic dysfunction-associated steatotic liver disease (MASLD) is characterized by the accumulation of fat in the liver, excluding excessive alcohol consumption and other known causes of liver injury. Animal models are often used to explore different pathogenic mechanisms and therapeutic targets of MASLD. The aim of this study is to apply an artificial intelligence (AI) system based on second-harmonic generation (SHG)/two-photon-excited fluorescence (TPEF) technology to automatically assess the dynamic patterns of hepatic steatosis in MASLD mouse models. Methods: We evaluated the characteristics of hepatic steatosis in mouse models of MASLD using AI analysis based on SHG/TPEF images. Six different models of MASLD were induced in C57BL/6 mice by feeding with a western or high-fat diet, with or without fructose in their drinking water, and/or by weekly injections of carbon tetrachloride. Results: Body weight, serum lipids, and liver enzyme markers increased at 8 and 16 weeks in each model compared to baseline. Steatosis grade showed a steady upward trend. However, the non-alcoholic steatohepatitis (NASH) Clinical Research Network (CRN) histological scoring method detected no significant difference between 8 and 16 weeks. In contrast, AI analysis was able to quantify dynamic changes in the area, number, and size of hepatic steatosis automatically and objectively, making it more suitable for preclinical MASLD animal experiments. Conclusions: AI recognition technology may be a new tool for the accurate diagnosis of steatosis in MASLD, providing a more precise and objective method for evaluating steatosis in preclinical murine MASLD models under various experimental and treatment conditions.

## 1. Introduction

Metabolic dysfunction-associated steatotic liver disease (MASLD), previously termed non-alcoholic fatty liver disease (NAFLD), is characterized by the accumulation of fat in the liver, excluding excessive alcohol consumption and other known causes of liver injury. MASLD includes simple steatosis, metabolic dysfunction-associated steatohepatitis (MASH), liver fibrosis, cirrhosis, and hepatocellular carcinoma [1,2,3]. MASLD is estimated to have a global prevalence of approximately 30% in the general population and maybe a major indication for liver transplantation [4,5,6]. The pathogenesis of MASLD is primarily a dysfunctional stress response to an excess supply of nutrient matrix in the liver, which promotes fibrosis and eventually leads to cirrhosis [7]. Therefore, treatment against lipid overload, insulin resistance, inflammation, and fibrosis have the potential to hinder the progression of MASLD development [8,9,10]. However, despite the recent conditional approval of resmetirom by the FDA, effective drug treatment for MASLD/MASH has been lacking [11,12]. Therefore, designing safe interventions for MASLD treatment is crucial.

The primary histological features of MASLD include steatosis, inflammation, hepatocyte ballooning, and fibrosis. Hepatic steatosis is the most important diagnostic feature of MASLD and is associated with the progression of steatohepatitis and fibrosis. Therefore, an accurate assessment of steatosis severity is directly related to the development of drugs for this disease. Animal models are often used to explore different pathogenic mechanisms and therapeutic targets of MASLD because of limitations in conducting drug trials in humans [13,14,15]. MASLD/MASH is mainly induced in mouse models through diet, chemical induction, gene modification, or a combination of these methods. However, limited published data exist on hepatic steatosis grading in animal models, and the Brunt or NASH Clinical Research Network (CRN) scoring systems are generally used as alternatives [16,17]. However, these systems are semi-quantitative histological grading methods that only assess the percentage of hepatic steatosis and do not reflect subtle changes and degrees of steatosis. Moreover, there is significant inter- and intra-reader variability assessment among pathologists with varying levels of experience. Therefore, an accurate, objective, and dynamic tool for evaluating the histology of preclinical MASLD animal models, particularly the degree of steatosis, is of great value for the development of more effective treatments.

Digital approaches for MASLD pathology assessment are reported, and images of stained tissue or second harmonic (SHG)-two-photon excited fluorescence (TPEF) images are mainly analyzed by laser scanning unstained slides [18,19,20,21]. The SHG/TPEF technique, supported by artificial intelligence (AI) analysis, has been used to evaluate steatosis and fibrosis in organs, including the liver [18,22,23]. Although the potential advantages of AI-enabled digital pathology have been recognized, there are currently few reports on the application of this automated assessment method in MASLD mouse models. Therefore, this study applied an AI system based on SHG/TPEF technology to automatically assess the dynamic patterns of hepatic steatosis in six MASLD mouse models.

## 2. Materials and Methods

### 2.1. Mouse Models of MASLD

Male C57BL/6 mice were purchased from Beijing Vital River Laboratory Animal Technology Co., Ltd. (Beijing, China). They were maintained at 25 °C with a 12 h light/dark cycle and allowed standard chow and water ad libitum until the time of the study. The Animal Experimental Ethics Committee of the Peking University People’s Hospital approved all the protocols used in this study (No. 2019PHC004). Western diet (21.2% fat, 17.3% protein, 48.5% carbohydrates based on caloric content; WD; TD120528) and 45% high-fat diet (41% fat, 24% protein, 24% carbohydrates based on caloric content; HFD; MD12032) were purchased from Medicine Ltd. (Yangzhou, China), and standard chow was provided by SPF Biotechnology Co., Ltd. (Beijing, China).

The mice were classified into six groups based on Western and HFDs, combined with or without fructose in water and with or without carbon tetrachloride (CCl_4_) injection, as reported in a previous study [24]. The six study groups were as follows: (1) WD-fed group; (2) WD-fed mice given high-fructose drinking water (23.1 g/L D-fructose and 18.9 g/L D-glucose) (WDF group); (3) WDF-fed mice given a weekly intraperitoneal injection of low-dose CCl_4_ (10%, 2.5 μL/g body weight) (WDF + CCl_4_); (4) HFD-fed group; (5) HFD-fed mice given high-fructose drinking water (HFDF); and (6) HFDF-fed mice given a weekly intraperitoneal injection of low dose CCl_4_ (HFDF + CCl_4_ group). Animal weights were recorded every 4 weeks. Mice were sacrificed at designated times (0, 8, and 16 weeks; *n* = 5 at each time point). Liver and blood were collected at the time of execution, and the wet weight of the liver was measured.

### 2.2. Blood Test

Blood samples were collected at indicated time points to measure serum alanine aminotransferase (ALT), aspartate aminotransferase (AST), total cholesterol (CHO), and low-density lipoprotein (LDL).

### 2.3. Liver Histopathology

Left lobe paraffin embedding and 4 μM continuous tissue sections were used for SHG/TPEF imaging, and conventional hematoxylin and eosin (H and E) staining was used to observe liver histological changes under an optical microscope. The histology of all mouse liver tissues was jointly and blindly assessed by two experts, and consensus scores were determined according to Brunt staging [16]. Steatosis was classified into four grades (0–3), ranging from 0 (<5% hepatic steatosis) to 3 (>66% hepatic steatosis).

### 2.4. AI Analysis of Hepatic Steatosis

Images of unstained liver samples were collected using the SHG/TPEF imaging device Genesis (HistoIndex, Singapore). Collagen was visualized using SHG microscopy, and steatosis distribution was visualized using TPEF microscopy. Laser excitation at 780 nm was used, and SHG and TPEF signals were recorded at 390 nm and 550 nm, respectively. In previous studies, the same imaging setting has been tested to effectively excite tissue to produce SHG and autofluorescence signals [23,25]. Especially in the TPEF channel, fat vacuoles, which were occupied by lipid droplets, appear dark since there are no fluorescent molecules, such as NAD (P) H and flavins [26,27]. Image tiles were captured at 20× magnification with a resolution of 512 × 512 pixels per tile, with each tile covering 200 × 200 μm^2^. Multiple adjacent tiles were used to cover the entire tissue area of each slide [23].

**Figure 1:**
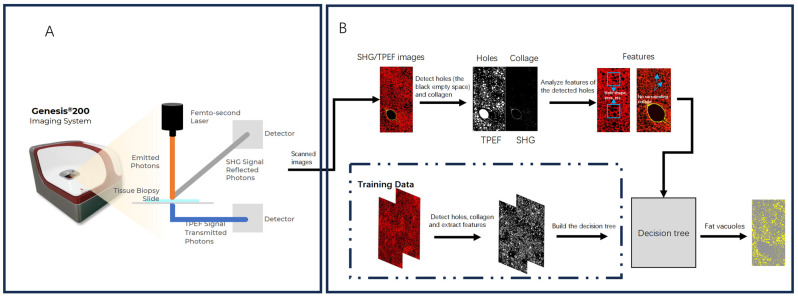
Flowchart of the imaging process and detection of fat vacuoles. (**A**) Images of unstained liver tissue samples were obtained using an SHG/TPEF imaging device (Genesis ^®^ 200). (**B**) All holes in the input images were detected in the TPE channel and classified using a pre-trained decision tree.

The SHG/TPEF images were analyzed using image processing and an AI-based algorithm that can detect steatosis features in various regions of liver tissue. (Figure 1) First, we preprocessed the TPEF channel with Otsu’s automatic threshold method to detect all empty holes in tissues and evaluate the severity of hepatic steatosis [28]. This method effectively segments images, helping us identify the areas corresponding to fat vacuole candidates by selecting an optimal threshold to highlight these empty holes. Second, because of the relatively low dimensionality of the feature data, we utilized a Classification and Regression Trees (CART) model which does not need pre-normalization, instead of other more sophisticated AI methods like neural networks to differentiate fat vacuoles from other vacuoles in the tissue [29], using the characteristics measured from these vacuole candidates such as intensity, area, circularity, density, and length-to-width ratio of vacuoles as well as the area of collagen surrounding those vacuoles, the model structure is shown in Figure S1. For example, fat vacuoles have more regular shapes and less surrounding collagen than the lumen of vessels, whose walls are full of structural collagen. Since hundreds of thousands of fat vacuole candidates could be in each sample, we trained the model with one tile ROI (200 × 200 μm^2^) from each of the 75 samples and applied this CART model to classify fat vacuoles in whole images. The same image analysis system was used to categorize liver samples into four histological regions: central vein, portal tract, perisinusoidal, and lobular area. A total of 45 steatosis parameters were calculated [23], including the percentage of steatosis (% area), number of fat vacuoles (# bubbles), and combinations of the size, number, and percentage of fat vacuoles associated with histological regions.

### 2.5. Statistical Analysis

Measurement data are expressed as the mean ± standard deviation. We compared the means between multiple groups using a one-way ANOVA test, and the Kolmogorov–Smirnov (KS) test was used to test the distribution of lipid droplet diameters. Statistical significance was set at *p* < 0.05.

## 3. Results

### 3.1. Liver Weight and Biochemical Characteristics of Different Mouse Models

**Figure 2:**
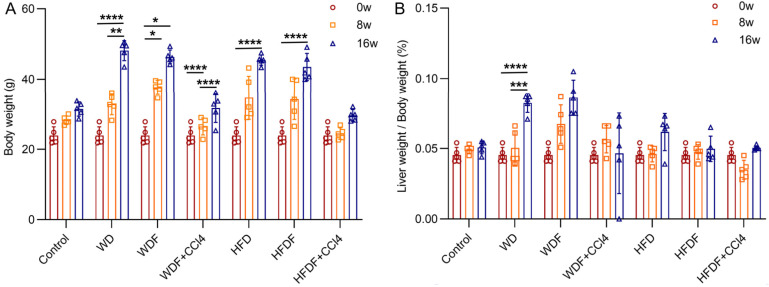
Average body weight (**A**) and liver weight-to-body weight ratio (**B**) of the control group and six MASLD mouse models at different time points (0, 8, and 16 weeks; *n* = 5 at each time point). Note: *, *p* < 0.05; **, *p* < 0.01; ***, *p* < 0.001; ****, *p* < 0.0001; w, week; CCl4, Carbon tetrachloride; WD, Western diet; WDF, WD with high-fructose drinking water; WDF + CCl_4_, WDF plus intraperitoneal injection of CCl_4_; HFD, high-fat diet; HFDF, HFD with high-fructose drinking water; HFDF + CCl_4_, HFD plus intraperitoneal injection of CCl_4_.

After induction for 8 and 16 weeks, mouse body weight and liver/body weight ratio in the different model groups increased compared to values at week 0, especially in the WD and WDF groups (Figure 2; *p* < 0.001).

**Figure 3:**
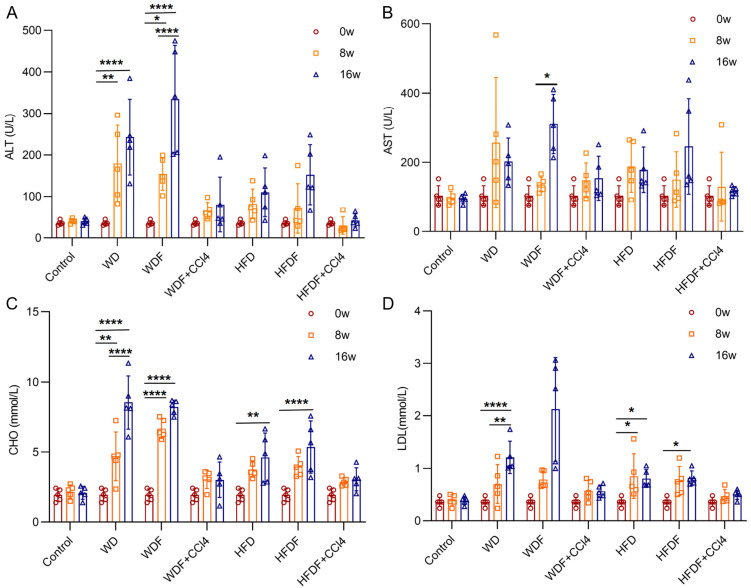
Serum levels of ALT (**A**), AST (**B**), cholesterol (CHO) (**C**), and low-density lipoprotein (LDL) (**D**) at different time points (0, 8, and 16 weeks) in the control group and six MASLD mouse models (*n* = 5 at each time point). Note: *, *p* < 0.05; **, *p* < 0.01; ****, *p* < 0.0001; w, week; CCl_4_, Carbon tetrachloride; WD, Western diet; WDF, WD with high-fructose drinking water; WDF + CCl_4_, WDF plus intraperitoneal injection of CCl_4_; HFD, high-fat diet; HFDF, HFD with high-fructose drinking water; HFDF + CCl_4_, HFD plus intraperitoneal injection of CCl_4_.

The serum levels of ALT, AST, CHO, and LDL in mice at 8 and 16 weeks were higher than those at 0 weeks, especially in the WD and WDF groups. However, in the mouse models administered CCl_4_, there was no significant difference in the levels of CHO and LDL at different time points (Figure 3).

### 3.2. Steatosis Features of the MASLD Mouse Models Using H and E Staining

**Figure 4:**
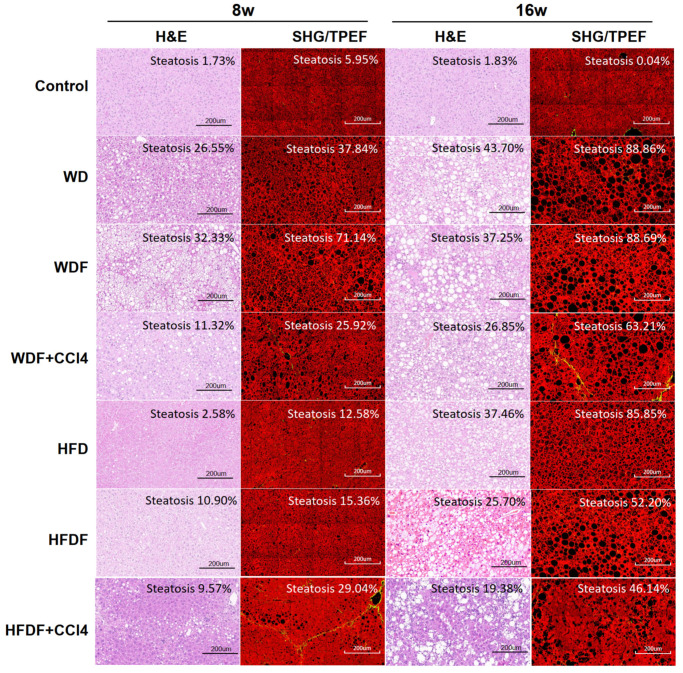
Representative images of H and E staining and SHG/TPEF at 8 and 16 weeks in the control group and six MASLD mouse models. In the H and E staining image, the percentages of vacuole area were shown as the percentages of steatosis, while in the SHG/TPEF image, the red channel represents TPEF, and the green channel represents SHG (collagen structure); the percentages of black fat vacuoles and surrounding affected areas were identified as the percentages of steatosis. H and E, Hematoxylin and eosin; SHG/TPEF, second-harmonic generation/two-photon-excited fluorescence; w, week; CCl_4_, Carbon tetrachloride; WD, Western diet; WDF, WD with high-fructose drinking water; WDF + CCl_4_, WDF plus intraperitoneal injection of CCl_4_; HFD, high-fat diet; HFDF, HFD with high-fructose drinking water; HFDF + CCl_4_, HFD plus intraperitoneal injection of CCl_4_; Bar: 200 μm.

Steatosis can be classified as macrovesicular (large lipid droplets) or microvesicular (small lipid droplets) [18]. After 8 weeks, all WD- and WDF-fed mice exhibited extensive micro- and macro-vesicular steatosis in zone 3 of the liver, whereas some HFD- and HFDF-fed mice did not exhibit these features until 16 weeks of treatment, at which point significant micro- and macro-vesicular steatosis was observed in 80–90% of hepatocytes across all lobules region in all mouse models (Figure 4). In the CCl_4_-treated groups (WDF + CCl_4_ and HFDF + CCl_4_), macrovesicular steatosis was the main steatotic feature, and steatosis was milder than that in the other groups (Figure 4). Compared to mice at 0 weeks, at 8 and 16 weeks, the grade of steatosis was significantly increased according to the NASH CRN score assessment. However, there was no significant difference in the grading of steatosis between the 8 and 16-week time points using this semi-quantified and subjective scoring approach (Table 1).

### 3.3. Steatosis Features in MASLD Mouse Models Using SHG/TPEF Imaging

**Figure 5:**
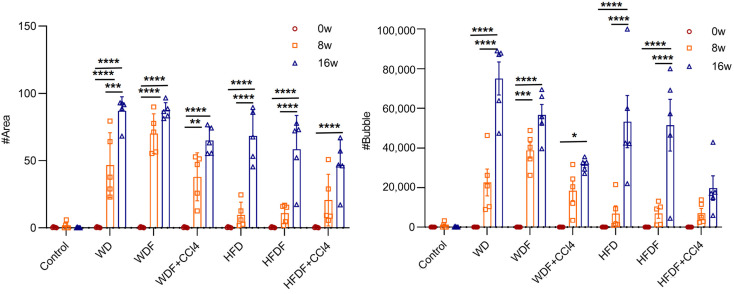
Steatosis quantification in the control group and six MASLD mouse model groups at different time points (0, 8, and 16 weeks). Quantitative parameters of steatosis (fat vacuoles and affected cell area) based on SHG/TPEF images. Note: *, *p* < 0.05; **, *p* < 0.01; ***, *p* < 0.001; ****, *p* < 0.0001; the number of samples in each group was 5; w, week; CCl_4_, Carbon tetrachloride; WD, Western diet; WDF, WD with high-fructose drinking water; WDF + CCl_4_, WDF plus intraperitoneal injection of CCl_4_; HFD, high-fat diet; HFDF, HFD with high-fructose drinking water; HFDF + CCl_4_, HFD plus intraperitoneal injection of CCl_4_.

**Figure 6:**
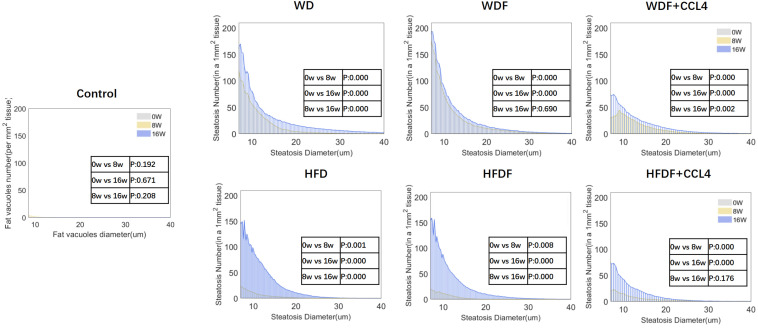
Fat vacuole distribution at different time points (0, 8, and 16 weeks) among the control group and six MASLD mouse models. The x-axis represents the diameter of the fat vacuoles (µm), whereas the y-axis represents the number of fat vacuoles per unit area (mm^2^), corresponding to the diameter of the fat vacuoles. Note: The comparison between different weeks of the same model is based on the difference in fat vacuole distribution according to their diameter per unit area. The *p*-value of the KS test is shown in the figure. w, week; CCl_4_, Carbon tetrachloride; WD, Western diet; WDF, WD with high-fructose drinking water; WDF + CCl_4_, WDF plus intraperitoneal injection of CCl_4_; HFD, high-fat diet; HFDF, HFD with high-fructose drinking water; HFDF + CCl_4_, HFD plus intraperitoneal injection of CCl_4_.

AI was used to identify and quantify the areas of steatosis using SHG/TPEF non-staining imaging to objectively assess the progression of hepatic steatosis (Figure 5). The results showed that steatosis in the liver was significantly increased after 8 and 16 weeks of treatment. According to the AI analysis, the percentage of intrahepatic fatty vacuole area (% Area) and the number of fatty vacuoles (# Bubble) significantly increased with increasing treatment time (Figure 5). Compared to that at 0 weeks, the diameter of fat vacuoles in the liver in each model increased with time (8 and 16 weeks), and the number of fat vacuoles per unit area at a given fat vacuole diameter also showed an upward trend. The most significant increase was observed in the WD and WDF groups (Figure 6). Although the increase in fat vacuoles in the HFD and HFDF groups was smaller at 8 weeks, it was greater at 16 weeks. After CCl_4_ administration (WDF + CCl_4_ and HFDF + CCl_4_), the experimental results showed that at the same time point (8 or 16 weeks), the quantitative levels of % Area and # Bubbles (Figure 5) were lower than those in the other diet-only groups. There was a significant decrease in the number of fat vacuoles per mm^2^ of tissue (Figure 5). These phenomena indicate that the increase in the number of fat vacuoles after the introduction of CCl_4_ was significantly reduced. Therefore, using AI, a quantified and objective method, we observed significant differences in the diameter and number of liver fat vacuoles between 8 and 16 weeks in most MASLD mouse models.

In addition, we introduced the results based on Hematoxylin and eosin (H and E) staining AI analysis to verify the accuracy of the non-stained AI analysis (Figure S2 and Table 1). The staining results showed that the percentage of intrahepatic fatty vacuole area (% r_Vacuole Area) and the number of fatty vacuoles (# Vacuole Count) significantly increased with time (8 and 16 weeks), as shown in Figure S2. After the introduction of CCl_4_, both models showed a corresponding decrease in the number of fat vacuoles. These phenomena are generally consistent with the unstained results, and we provide a detailed description of the inter-group comparison results for weeks 0, 8, and 16 in Table S1. Furthermore, we compared the correlation analysis results between AI and traditional pathology, where the correlation between the non-stained AI analysis results in % Area and # Bubble was 0.939 and 0.939, and the correlation between the stained AI analysis results in % r_Vacuole Area and #Vacuole Count was 0.882 and 0.828, respectively, with *p* values less than 0.05 (Table S2).

## 4. Discussion

Although mouse models are commonly used to study MASLD mechanisms and develop new drugs, research on fully automated and objective methods for estimating hepatic steatosis in mouse models is limited. In mouse models, MASLD is typically induced by diet, chemicals, genetic modifications, or a combination of these factors. In this study, six models were developed using either diet alone or a combination of diet, high-fructose drinking water, and CCl_4_. An automated AI evaluation system based on SHG/TPEF imaging was used to analyze the characteristics of hepatic steatosis, and it was suggested that this AI system was superior to traditional grading evaluations of pathological steatosis.

MASLD is mainly characterized by disturbances in lipid metabolism in the liver, resulting in triglyceride accumulation. Fat accumulation in the liver is a hallmark of MASLD. The extent of steatosis can be associated with inflammation and is typically one of several factors contributing to MASH. In the early stages of the disease, steatosis is an upstream event that reflects metabolic disorders leading to cell damage, inflammation, and fibrosis [18,30,31]. Therefore, the accurate measurement of steatosis is important for determining the degree of MASLD progression. However, only a few studies have used animal models for the automatic diagnosis of MASLD. Acorda et al. [32] and Starke et al. [33] analyzed the automatic evaluation of liver ultrasound images to diagnose fatty liver in cows. Peng et al. [34] evaluated the effects of dual-echo Dixon in-phase and out-of-phase, chemical shift imaging (CSI), and hydrogen magnetic resonance spectroscopy (H-MRS) methods on the determination of liver fat content in mice. They concluded that CSI and MRS have advantages for evaluating liver fat changes. Cao et al. [35] used 1H-MRS and CT liver imaging techniques to quantify the absolute intrahepatic lipid levels, suggesting the feasibility of evaluating mouse steatosis. However, despite being widely used, the diagnostic accuracy of ultrasound for mild steatosis is low, and MRS is complex and expensive. Liver tissue examination remains the gold standard for accurate evaluation of liver steatosis.

Ge et al. [36] were the first to use digital pathology to evaluate steatosis patterns in the mouse liver, describing the digital image obtained through automatic recognition and the recognition area stained with oil red O. De Rudder et al. [37] showed that an automated measurement method for fatty degeneration based on quantification of the area of hepatic bullous steatosis significantly correlated with CT liver density, fat content, steatosis score, and CD36 expression. This automated tool can be used to identify and quantify bullous steatosis accurately. Ramot et al. [38] compared an evaluation based on artificial semi-quantitative microscopy with the data output of artificial intelligence software, which can accurately diagnose the percentage of liver fat vesicles in MASLD mice. However, these methods rely on stained slices, and the heterogeneity of stained slices may affect the accuracy of the machine interpretation.

SHG/TPEF is a computer-assisted microscopic imaging technique that allows for image acquisition of tissue structural features in an unstained section. Our previous studies [23] and others [25] suggested that machine learning based on SHG/TPEF can be used to analyze liver steatosis. Due to the lack of fluorescent molecules, cell nuclei and degenerated hepatocyte regions could also appear darker besides lipid droplets in the TPEF channel. However, The size of nuclei is much smaller than macrosteatotic fat vacuole, and even though it could look similar to microsteatotic fat vacuole, the number of nuclei (one per hepatocyte) is much less compared with the number of those small lipid droplets (dozens per hepatocyte). Therefore, the impact of misidentifying cell nuclei as microsteatotic fat vacuoles, if any, is very limited. Besides, degenerated hepatocytes are not fully dark since there are more or less fluorescent molecules left; on the other hand, fat vacuoles previously occupied by lipid droplets have no fluorescent molecules and, therefore, no autofluorescence signals at all, making the boundary of fat vacuoles very clear to measure their diameters. In this study, AI analysis based on SHG/TPEF images not only showed a significant increase in the number of fat vacuoles in all six MASLD mouse models but also showed an upward trend in the area, number, and diameter of fat vacuoles in each group, as well as the number of fat droplets per unit area under the corresponding fat vacuole diameter, with a significant difference between 8 and 16 weeks. Although the NASH CRN score was used to determine the degree of steatosis, there was no significant difference between 8 and 16 weeks. The findings suggest that the liver AI automated evaluation system can dynamically observe the area, number, and size characteristics of hepatic steatosis and analyze its changes quantitatively, which is superior to the semi-quantitative scoring evaluation of steatosis.

Analysis of the distribution characteristics of steatosis changes suggested that they occurred earlier in the WD and WDF mouse models than in other models. The administration of CCl_4_ delayed the occurrence of steatosis, which may be related to its liver toxicity. The observation that CCl_4_ administration affects steatosis timing is consistent with the findings of Tsuchida et al. and Zhang et al. [39,40], in which the addition of CCl_4_ to WD reduced weight gain in mice. In preclinical trials, quantitative analysis of the different characteristics of steatosis may improve the accuracy of the evolution and regression assessment of MASLD. Dietary-induced models and gene-modified models could be appropriately selected based on dynamic pathological changes and experimental purposes.

Although hepatocytes in MASLD may contain large droplets of macrovesicular fat, mixed large and small droplets may also appear. With the development of cirrhosis, the extent of steatosis decreases [41], but the clinical significance of steatosis requires further study. AI can be used to analyze the differences in the progression of steatosis in MASLD and associated cirrhosis. Furthermore, AI-based digital images can provide more reproducible, objective, and accurate data for disease progression and treatment monitoring.

This study had some limitations. First, this study used only male mice; differences may have arisen if female mice were included. Second, water balance or intra-abdominal fat was not detected in animal models, and the physiologic change in fat could not be refeeled. Norihiro Kubota et al. demonstrated that there were no differences in visceral fat or liver weights between the normal diet and HFD CCl_4_ groups [42]. Thirdly, several methods for generating automated quantitative readings of steatosis have been translated into commercial platforms using AI-based digital pathology methods. However, there are no publicly available data on the consistency and cross-instrument consistency of these platforms [18]. In the future, the validation of steatosis across instrument results should be performed and standardized.

## 5. Conclusions

In this study, we established six MASLD mouse models. AI-based quantitative evaluation of SHG/TPEF images could obtain novel insights on steatosis progression by analyzing the size and distribution characteristics of steatosis in different models. Therefore, the analysis using AI based on SHG/TPEF image could determine the degree and distribution of steatosis in the liver of MAFLD mice, avoiding intraobserver and interobserver variability. AI recognition technology may be a new tool to accurately diagnose steatosis in MASLD, providing a more precise and objective method for evaluating steatosis in preclinical murine MASLD models under various experimental and treatment conditions.

## Figures and Tables

**Table 1:** Steatosis grade of mice at different time points (0, 8, and 16 weeks) in the control group and six MAFLD mouse models.

	Control	WD	WDF	WDF + CCl_4_	HFD	HFDF	HFDF + CCl_4_
0 week	0.0 ± 0.0	0.0 ± 0.0	0.0 ± 0.0	0.0 ± 0.0	0.0 ± 0.0	0.0 ± 0.0	0.0 ± 0.0
8 weeks	0.0 ± 0.0	2.0 ± 1.4 ***	3.0 ± 0.0 ***	1.6 ± 0.5 **	1.4 ± 1.1 *	1.6 ± 0.9 *	0.8 ± 0.5
16 weeks #	0.0 ± 0.0	3.0 ± 0.0 ***	3.0 ± 0.0 ***	2.4 ± 0.9 ***	2.2 ± 0.4 **	2.2 ± 0.8 **	1.0 ± 0.7 *

Note: 8/16 weeks vs. 0 weeks, * *p* < 0.05; ** *p* < 0.01; *** *p* < 0.001; # 16 vs. 8 weeks all *p* > 0.05. CCl_4_, Carbon tetrachloride; WD, western diet; WDF, WD with high fructose drinking water; WDF + CCl_4_, WDF plus intraperitoneal injection of CCl_4_; HFD, high-fat diet; HFDF, HFD with high-fructose drinking water; HFDF + CCl_4_, HFD plus intraperitoneal injection of CCl_4_.

## Data Availability

The deidentified participant data will be from the corresponding author (F.L.) upon reasonable request.

## Supplementary Materials

The following supporting information can be downloaded at: https://www.mdpi.com/article/10.3390/diagnostics14242889/s1, Figure S1: Architecture of the decision tree model trained with CART. Node x1 represents area; x2 represents length-to-width ratio; x3 represents circularity; x4 represents intensity; x5 represents the area of collagen surrounding/area; x6 represents density; of vacuole candidates. All nodes are unitless except x1 (pixel). 1s in leaves mean fat vacuoles, 0 s otherwise. Figure S2: Quantitation of the percentage of vacuole area and vacuole count based on H&E staining image in the control group and six MAFLD mouse model groups at different time points (0, 8, and 16weeks). Note: *, *p* < 0.05; **, *p* < 0.01; ***, *p* < 0.001; ****, *p* < 0.0001; The number of samples in each group is 5; w, week; CCl_4_, Carbon tetrachloride; WD, western diet; WDF, WD with high fructose drinking water; WDF+CCl_4_, WDF plus intraperitoneal injection of CCl_4_; HFD, high-fat diet; HFDF, HFD with high-fructose drinking water; HFDF+CCl_4_, HFD plus intraperitoneal injection of CCl_4_. Table S1: Comparison of quantitative steatosis between non-staining SHG/TPEF (%Area and #Bubble) and H&E staining image (%Vacuole area and #Vacuole count). Table S2: Correlation analysis between AI analysis and traditional pathological scoring.
