# Supplementary material for: Hepatic Steatosis Analysis in Metabolic Dysfunction-Associated Steatotic Liver Disease Based on Artificial Intelligence
Source: Diagnostics (Basel). 2024 Dec 23;14(24):2889. doi: 10.3390/diagnostics14242889 (PMC11675354; doi:10.3390/diagnostics14242889)
Supplement: Supplementary file 1 [file diagnostics-14-02889-s001.zip › diagnostics-3335182-supplementary.pdf]

Supplementary Material:

Automated H&E staining section analysis

We compared the quantitative results of steatosis in both unstained and stained images, the AI-based method described in the main text was used to analyze fat vacuoles in unstained images, while in stained images, we used the HALO® Vacuole Quantification module to measure the entire sample, the software solution allows accurate discrimination of empty vacuole (size > 10μm2) (steatosis) contained in the entire liver sections and automatically discard the main vessels and large empty space. This program assesses automatically the number and the area of vesicles scattered in the entire liver section from the native RGB image (pixel size: 0.2738μm). The percentage of vacuole area and vacuole count were calculated.as shown in supplementary figure 1, the inter-group comparison results for each model within 0, 8 and 16 weeks were shown in supplementary table 1. In addition，we compared the lipid accumulation areas quantified by these two methods and their correlations with pathological scores, as shown in supplementary table 2. Both methods demonstrate good correlations with pathological scores, with the unstained method showing a higher correlation.

Supplementary Figure S1: Architecture of the decision tree model trained with CART. Node x1 represents area; x2 represents length-to-width ratio; x3 represents circularity; x4 represents intensity; x5 represents the area of collagen surrounding / area; x6 represents density; of vacuole candidates. All nodes are unitless except x1(pixel). 1s in leaves mean fat vacuoles, 0s otherwise.

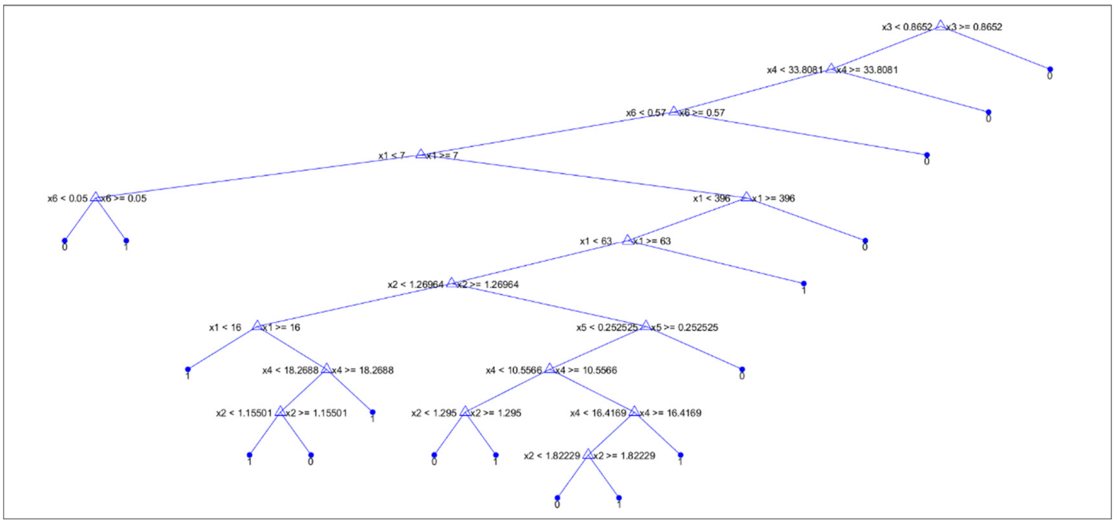

Supplementary Figure S2: Quantitation of the percentage of vacuole area and vacuole count based on H&E staining image in the control group and six MAFLD mouse model groups at different time points (0, 8, and 16weeks). Note: *, $P < 0.05$; **, $P < 0.01$; ***, $P < 0.001$; ****, $P < 0.0001$; The number of samples in each group is 5; w, week; CCl4, Carbon tetrachloride; WD, western diet; WDF, WD with high fructose drinking water; WDF+CCl4, WDF plus intraperitoneal injection of CCl4; HFD, high-fat diet; HFDF, HFD with high-fructose drinking water; HFDF+CCl4, HFD plus intraperitoneal injection of CCl4.

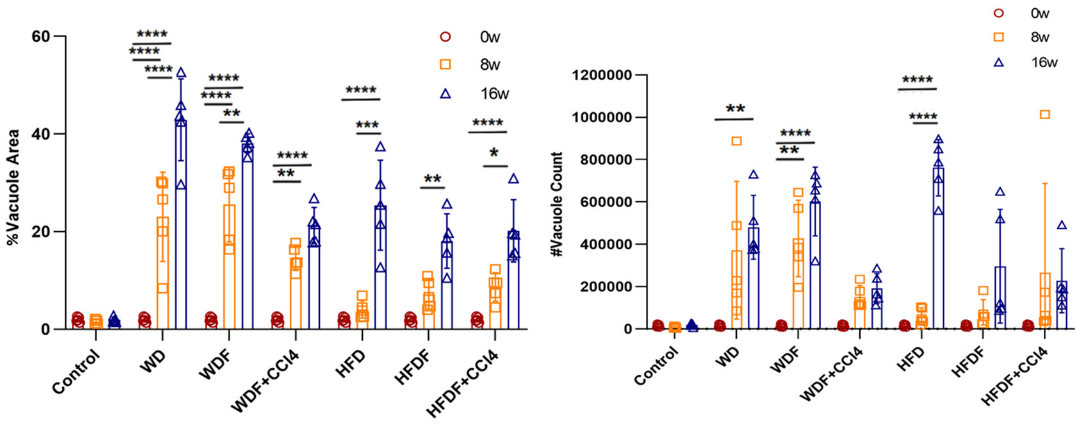

Supplementary Table S1: Comparison of quantitative steatosis between non-staining SHG/TPEF (%Area and #Bubble) and H&E staining image (%Vacuole area and #Vacuole count)

	Group	Mean Diff.	95.00% CI of diff.	Statistical significance
SHG/TPEF:%Area	Control:0w vs. Control:8w	-1.26	-30.17 to 27.65	ns
	Control:0w vs. Control:16w	0.2031	-28.70 to 29.11	ns
	Control:8w vs. Control:16w	1.463	-27.44 to 30.37	ns
	WD:0w vs. WD:8w	-46.29	-75.20 to -17.39	****
	WD:0w vs. WD:16w	-86.57	-115.5 to -57.67	****
	WD:8w vs. WD:16w	-40.28	-69.19 to -11.37	***
	WDF:0w vs. WDF:8w	-69.68	-98.58 to -40.77	****
	WDF:0w vs. WDF:16w	-86.85	-115.8 to -57.95	****
	WDF:8w vs. WDF:16w	-17.17	-46.08 to 11.73	ns
	WDF+CCl4:0w vs. WDF+CCl4:8w	-37.59	-66.49 to -8.683	**
	WDF+CCl4:0w vs. WDF+CCl4:16w	-64.57	-93.48 to -35.67	****
	WDF+CCl4:8w vs. WDF+CCl4:16w	-26.98	-55.89 to 1.923	ns
	HFD:0w vs. HFD:8w	-9.154	-38.06 to 19.75	ns
	HFD:0w vs. HFD:16w	-67.94	-96.84 to -39.03	****
	HFD:8w vs. HFD:16w	-58.78	-87.69 to -29.88	****
	HFDF:8w vs. HFDF:16w	-47.58	-76.48 to -18.67	****
	HFDF+CCl4:0w vs. HFDF+CCl4:8w	-20.27	-49.18 to 8.636	ns
	HFDF+CCl4:0w vs. HFDF+CCl4:16w	-46.43	-75.34 to -17.53	****

	HFDF+CCl4:8w vs. HFDF+CCl4:16w	-26.16	-55.07 to 2.744	ns
SHG/TPEF:#Bubble	Control:0w vs. Control:8w	-866.6	-28529 to 26796	ns
	Control:0w vs. Control:16w	-32.8	-27695 to 27630	ns
	Control:8w vs. Control:16w	833.8	-26829 to 28496	ns
	WD:0w vs. WD:8w	-22528	-50190 to 5135	ns
	WD:0w vs. WD:16w	-75035	-102697 to -47373	****
	WD:8w vs. WD:16w	-52507	-80170 to -24845	****
	WDF:0w vs. WDF:8w	-38801	-66463 to -11138	***
	WDF:0w vs. WDF:16w	-56636	-84299 to -28974	****
	WDF:8w vs. WDF:16w	-17836	-45498 to 9827	ns
	WDF+CCl4:0w vs. WDF+CCl4:8w	-18358	-46020 to 9305	ns
	WDF+CCl4:0w vs. WDF+CCl4:16w	-31424	-59086 to -3761	*
	WDF+CCl4:8w vs. WDF+CCl4:16w	-13066	-40728 to 14596	ns
	HFD:0w vs. HFD:8w	-6876	-34538 to 20787	ns
	HFD:0w vs. HFD:16w	-53268	-80930 to -25606	****
	HFD:8w vs. HFD:16w	-46392	-74055 to -18730	****
	HFDF:0w vs. HFDF:8w	-7014	-34676 to 20649	ns
	HFDF:0w vs. HFDF:16w	-51472	-79134 to -23809	****
	HFDF:8w vs. HFDF:16w	-44458	-72120 to -16796	****
	HFDF+CCl4:0w vs. HFDF+CCl4:8w	-7099	-34762 to 20563	ns
	HFDF+CCl4:0w vs. HFDF+CCl4:16w	-19723	-47385 to 7939	ns

	HFDF+CCl4:8w vs. HFDF+CCl4:16w	-12624	-40286 to 15039	ns
H&E:%Vacuole area	Control:0w vs. Control:8w	0.4106	-9.970 to 10.79	ns
	Control:0w vs. Control:16w	0.04395	-10.34 to 10.42	ns
	Control:8w vs. Control:16w	-0.3667	-10.75 to 10.01	ns
	WD:0w vs. WD:8w	-21.01	-31.39 to -10.63	****
	WD:0w vs. WD:16w	-40.9	-51.28 to -30.52	****
	WD:8w vs. WD:16w	-19.89	-30.27 to -9.509	****
	WDF:0w vs. WDF:8w	-23.54	-33.92 to -13.16	****
	WDF:0w vs. WDF:16w	-36.06	-46.44 to -25.68	****
	WDF:8w vs. WDF:16w	-12.53	-22.91 to -2.147	**
	WDF+CCl4:0w vs. WDF+CCl4:8w	-12.51	-22.89 to -2.128	**
	WDF+CCl4:0w vs. WDF+CCl4:16w	-19.25	-29.63 to -8.868	****
	WDF+CCl4:8w vs. WDF+CCl4:16w	-6.74	-17.12 to 3.641	ns
	HFD:0w vs. HFD:8w	-2.05	-12.43 to 8.330	ns
	HFD:0w vs. HFD:16w	-23.4	-33.78 to -13.01	****
	HFD:8w vs. HFD:16w	-21.34	-31.73 to -10.96	****
	HFDF:0w vs. HFDF+CCl4:0w	-5.33E-15	-10.38 to 10.38	ns
	HFDF:0w vs. HFDF+CCl4:8w	-6.649	-17.03 to 3.732	ns
	HFDF:8w vs. HFDF+CCl4:16w	-13.04	-23.42 to -2.658	**
	HFDF+CCl4:0w vs. HFDF+CCl4:8w	-6.649	-17.03 to 3.732	ns
	HFDF+CCl4:0w vs. HFDF+CCl4:16w	-18.15	-28.53 to -7.766	****

	HFDF+CCl4:8w vs. HFDF+CCl4:16w	-11.5	-21.88 to -1.117	*
H&E:#Vacuole count	Control:0w vs. Control:8w	8493	-348422 to 365408	ns
	Control:0w vs. Control:16w	-3199	-360114 to 353716	ns
	Control:8w vs. Control:16w	-11692	-368607 to 345223	ns
	WD:0w vs. WD:8w	-356459	-713375 to 455.9	ns
	WD:0w vs. WD:16w	-464743	-821658 to -107828	**
	WD:8w vs. WD:16w	-108284	-465199 to 248631	ns
	WDF:0w vs. WDF:8w	-412027	-768942 to -55112	**
	WDF:0w vs. WDF:16w	-586307	-943222 to -229392	****
	WDF:8w vs. WDF:16w	-174280	-531195 to 182635	ns
	WDF+CCl4:0w vs. WDF+CCl4:8w	-135252	-492167 to 221663	ns
	WDF+CCl4:0w vs. WDF+CCl4:16w	-174830	-531745 to 182086	ns
	WDF+CCl4:8w vs. WDF+CCl4:16w	-39577	-396493 to 317338	ns
	HFD:0w vs. HFD:8w	-45557	-402473 to 311358	ns
	HFD:0w vs. HFD:16w	-746210	-1103125 to -389295	****
	HFD:8w vs. HFD:16w	-700653	-1057568 to -343738	****
	HFDF:0w vs. HFDF:8w	-63180	-420095 to 293735	ns
	HFDF:0w vs. HFDF:16w	-280437	-637352 to 76478	ns
	HFDF:8w vs. HFDF:16w	-217257	-574173 to 139658	ns

	HFDF+CCl4:0w vs. HFDF+CCl4:8w	-250030	-606945 to 106885	ns
	HFDF+CCl4:0w vs. HFDF+CCl4:16w	-212371	-569286 to 144544	ns
	HFDF+CCl4:8w vs. HFDF+CCl4:16w	37659	-319256 to 394574	ns

Note: *ns ,P>0.05;* *, *P* < 0.05; **, *P* < 0.01； ***, *P* < 0.001; ****, P < 0.0001.

Supplementary Table S2: Correlation analysis between AI analysis and traditional pathological scoring

Spearman **correlation coefficient**					
	Steatosis Grade	%r_Vacuole Area(H&E)	#Vacuole Count(H&E)	%Area (SHG/TPEF)	#Bubble (SHG/TPEF)
Steatosis Grade	1.000	.882****	.828****	.939****	.939****
%r_Vacuole Area(H&E)	.882****	1.000	.885****	.899****	.887****
#Vacuole Count(H&E)	.828****	.885****	1.000	.805****	.822****
%Area (SHG/TPEF)	.939****	.899****	.805****	1.000	.966****
#Bubble (SHG/TPEF)	.939****	.887****	.822****	.966****	1.000

The comparison of steatosis-related parameters quantified by two methods. Steasis Grade is the grading of stained samples by pathologists, %r_Vacuole Area (H&E) and # Vacuole Count (H&E) are results obtained based on staining AI methods ,%Area(SHG/TPEF) and #Bubble(SHG/TPEF) are the results obtained based on non-staining AI methods. Note: *, $P < 0.05$; **, $P < 0.01$； ***, $P < 0.001$; ****, P < 0.0001.
